# TLR7 signaling aggravates lung inflammation associated with increased anti-Scl-70 autoantibody production in murine bleomycin-induced systemic sclerosis

**DOI:** 10.3389/fimmu.2026.1823229

**Published:** 2026-07-01

**Authors:** Jefferson Fernandes Evangelista, Ana Karina Nisperuza Vidal, Donghua Xu, Mohammad Islamuddin, Yilin Chen, Chenxiao Wang, Raul Freitas, Shumei Liu, Elizabeth Engler-Chiurazzi, Robert V. Blair, Prasun K. Datta, Xuebin Qin

**Affiliations:** 1 Division of Comparative Pathology, Tulane National Biomedical Center, Covington, LA, United States; 2 Department of Microbiology and Immunology, Tulane University School of Medicine, New Orleans, LA, United States; 3 Department of Neurosurgery and Neurology, Clinical Neuroscience Research Center, Tulane Brain Institute, Tulane University School of Medicine, New Orleans, LA, United States

**Keywords:** anti-Scl-70 antibodies, bleomycin, systemic sclerosis, TLR7, IRF7, CCL12, CCL2, CCR2

## Abstract

**Objective:**

Systemic sclerosis (SSc) is a severe autoimmune disease characterized by immune dysregulation, fibrosis, and substantial morbidity and mortality. Although type I interferon-related pathways have been implicated in SSc, the contribution of Toll-like receptor 7 (TLR7) and its downstream signaling, including the transcription factor interferon regulatory factor 7 (IRF7) and effector molecules such as CCL2 and CCL12, to disease pathogenesis remains unclear.

**Methods:**

We used a bleomycin (BLM)-induced mouse model of SSc. Male wild-type (WT), *Tlr7*-deficient (*Tlr7^−^/^−^*), and *Irf7*-deficient (*Irf7^−^/^−^*) mice (10–12 weeks old) received daily subcutaneous BLM (2.5 mg/kg/day) for 4 weeks. In a separate experiment, BLM-treated WT mice were treated with the CCR2 antagonist (RS504393). Disease features were evaluated by histopathology, ELISA, flow cytometry, western blotting, RT-qPCR, and Olink proteomics.

**Results:**

BLM-treated *Tlr7^−^/^−^* and *Irf7^−^/^−^* mice showed less body weight loss, reduced pulmonary interstitial inflammation, fewer inflammatory monocytes in the spleen, lower pulmonary type I interferon-related gene expression, and lower serum anti-topoisomerase I autoantibody (anti-Scl-70) levels. Pharmacologic CCR2 antagonism attenuated BLM-induced body weight loss, lung injury, and reduced dermal collagen deposition.

**Conclusion:**

These findings support a pathogenic role for TLR7-IRF7-IFN-I signaling in anti-Scl-70 autoantibody production and pulmonary inflammation in experimental SSc, and suggest that downstream chemokine pathways may represent therapeutic targets.

## Introduction

Systemic sclerosis (SSc) is a rare autoimmune disease characterized by immune dysregulation, vasculopathy, and progressive fibrosis of the skin and lungs (1). Despite its low prevalence, SSc has the highest mortality rate among rheumatic diseases. Pulmonary complications are severe, with 50–65% of patients exhibiting interstitial lung abnormalities, and up to 35% of SSc deaths caused by interstitial lung disease (ILD) (2, 3). Anti-topoisomerase I (anti-Scl-70) autoantibodies are associated with diffuse cutaneous SSc, severe skin fibrosis, rapidly progressive lung disease, and poorer outcomes (4–6). Although SSc is more common in women, men may present with more severe lung involvement and worse outcomes in some cohorts (7). However, how innate and adaptive immune responses interact to drive ILD progression in SSc remains incompletely understood (8).

Among endosomal Toll-like receptors (TLRs), TLR7 recognizes single-stranded RNA (ssRNA) and signals through the adaptor MyD88, leading to activation of two major downstream pathways: NF-κB, which promotes pro-inflammatory cytokine production, and interferon regulatory factor 7 (IRF7), the master transcription factor driving type I interferon (IFN-I) responses (9). IRF7 coordinates the production of IFN-α and IFN-β, particularly in myeloid cells (10). In SSc, a prominent IFN-I gene signature in blood and affected tissues has been associated with disease severity and organ involvement (11–13). Human gain-of-function variants in TLR7 have been linked to systemic autoimmunity, characterized by broad B-cell hyperactivation, abnormal autoantibody production, and multi-organ inflammation, highlighting the pathogenic relevance of this pathway (14, 15). Polymorphisms in IFN-related genes, including IRF7, have also been linked to increased susceptibility to disease (16). TLR7 expression is increased in the skin of SSc patients compared with healthy controls (17). Activation of the IFN-I pathway in SSc has been associated with severe organ involvement and poor prognosis (18). Abnormal TLR7 activation has been shown to drive autoreactive B cells and autoantibody production in lupus-prone mice (19). Given the central role of TLR7 and IRF7 in IFN-I–driven immune activation, this axis may represent a relevant therapeutic target in SSc (20). Importantly, beyond cytokine and interferon production, activation of this pathway also contributes to the recruitment of immune cells to inflamed tissues.

Monocyte recruitment is a key component of inflammatory and fibrotic responses in SSc, particularly in the lung (21). Among the mediators involved, CCL2 (MCP-1) and CCL12 (mouse MCP-5) play central roles in CCR2-dependent monocyte trafficking. Consistent with this, TLR7 activation has been shown to induce chemokine programs that amplify leukocyte recruitment, including CCL2/CCL12 in mice and CCL2 in humans (22, 23). These chemokines are of particular interest because monocyte and macrophage recruitment is thought to contribute to persistent inflammation and tissue remodeling in SSc (24, 25). CCL2 and its murine homolog CCL12 are key ligands for CCR2 that promote monocyte mobilization from the bone marrow and recruitment to inflamed tissues, thereby linking innate immune activation to chronic inflammation and fibrosis (26–28). Clinical studies also indicate that increased CCL2 is associated with high anti-topoisomerase or anti-RNA polymerase I/III antibody reactivity, and with greater frequency of major organ-based complications (29). Suppression and blockage of CCL2 functionality has shown to attenuate disease severity in SSc in mice (26, 30). However, the upstream signaling that modulates CCL2/CCL12 in SSc development remains unclear.

Bleomycin (BLM)-induced skin and lung fibrosis is a standard model for mechanistic and therapeutic studies of SSc (31). This model reproduces key inflammatory and fibrotic features of SSc, although it does not fully recapitulate the complexity of the human disease. A recent study has shown that TLR7 deficiency attenuates skin and lung fibrosis in BLM-induced SSc model and is associated with reduced inflammatory cell infiltration and lower expression of pro-inflammatory and pro-fibrotic mediators in affected tissues (32). Moreover, increased IRF7 expression has been observed in the skin and fibroblasts of SSc patients, and IRF7 deficiency in mice also reduces BLM-induced inflammation and dermal fibrosis (33). However, whether TLR7-IRF7 signaling contributes to lung pathology through downstream chemokine programs such as CCL2/CCL12 remains unclear. Because lung involvement is a major determinant of severe SSc, defining how TLR7 and IRF7 shape pulmonary inflammation could provide mechanistic insight and identify new therapeutic targets.

To address these questions, we used a BLM-induced mouse model of SSc in wild-type (WT), TLR7-deficient (*Tlr7^−/−^*), and IRF7-deficient (*Irf7^−/−^*) mice and characterized disease development across genotypes. We show that TLR7 or IRF7 deficiency attenuated BLM-induced SSc-like disease features in this model. This protection is associated with reduced infiltration of inflammatory monocytes in the spleen, decreased IFN-I gene expression in the lung, lower serum anti-Scl-70 levels, and reduced pulmonary levels of CCL2 and CCL12. Furthermore, antagonism of CCR2 reduced BLM-mediated body weight loss and lung injury. Together, these findings support a pathogenic role for TLR7-IRF7-IFN-I axis in pulmonary inflammation and autoantibody-associated disease manifestations and suggest that targeting downstream CCL2/CCL12-CCR2 signaling may represent a potential therapeutic strategy in SSc.

## Methods

### Animals

Male and female C57BL/6J (B6) mice (strain 000664; Jackson Laboratory, USA) were first used in pilot studies to determine the BLM formulation, dose, and treatment window that best generated a non-lethal SSc-like phenotype with skin and lung involvement (*n* = 3-5) (**Supplementary Figure 1**). For subsequent studies, 10- to 12-week-old male B6, as well as aged-matched *Tlr7^−/−^* (strain 008380; Jackson Laboratory) and *Irf7^−/−^* mice (RBRC01420; RIKEN, Japan), all on a B6 background, were used (*n* = 3-5). All procedures were approved by the Institutional Animal Care and Use Committee at Tulane University (protocol #2285).

### SSc induction and sample collection

BLM (APExBIO) was freshly reconstituted in sterile PBS at a concentration of 1 mg/mL and administered subcutaneously into the interscapular dorsal skin (2.5 mg/kg/day; 60–100 µL) once daily for 28 consecutive days. Control B6 mice received an equivalent volume of PBS. Experimental groups included BLM-treated WT, *Tlr7^−^/^−^*, and *Irf7^−^/^−^* mice. BW was monitored every other day throughout the treatment period. Mice were euthanized 24 h after the final BLM or PBS injection (day 29). This dose and treatment duration were selected based on pilot studies showing a non-lethal model with both skin and lung involvement and, importantly, permitting detection of genotype-dependent differences in disease severity. The blood was collected via cardiac puncture into serum tubes, allowed to clot for 30 min, and then centrifuged (1500 × g, 10 min, room temperature) to collect serum. Spleens were processed for flow cytometry. The right lung was gently inflated with buffered zinc formalin (Z-Fix^®^, Anatech) and fixed for histology and immunofluorescence, along with dorsal skin samples. The left lung and skin tissues were snap-frozen in liquid nitrogen for molecular assays. Flow cytometry, ELISA, H&E staining, immunostaining (antibodies listed in **Supplementary Table 1**), Western blotting (antibodies listed in **Supplementary Table 2**), qRT-PCR (primers listed in **Supplementary Table 3**), and Olink proteomics were performed as described in the **Supplementary Materials** and Methods under masked conditions.

### CCR2 antagonist treatment

RS504393 (MedChem Express; Cat. No. HY-15418) is a selective antagonist of the CCR2 chemokine receptor. The compound was administered to WT mice by oral gavage at 2 mg/kg/day for 28 consecutive days, based on previously published studies using systemic CCR2 inhibition (34). RS504393 was given 4 hours prior to each BLM injection to ensure CCR2 blockade during ongoing BLM-induced injury. Control mice (WT+BLM) received vehicle by oral gavage under the same schedule.

### Experimental rigor and statistical analysis

Group sizes ranged from 3 to 5 mice per group, depending on mouse availability, and the collected tissues were processed using the sample method or buffer for multiple downstream analyses. Final group sizes were WT+PBS (*n* = 3), WT+BLM (*n* = 5), *Tlr7^−^/^−^*+BLM (*n* = 5), and *Irf7^−^/^−^*+BLM (*n* = 5) for mouse body weight change, skin thickness, serum antibody and cytokine, and spleen immune cell analyses. However, for the lung-related assays, we used only four lung samples per group because these lungs were prepared using the same method and buffer for lung H&E staining, Masson’s trichrome staining, lung qRT-PCR, and Olink proteomic analyses. No formal *a priori* power calculation was performed. The exact *n* for each group is reported in the figure legends. Randomization was performed by allocating mice to their corresponding genotypic groups once the required number of age-matched, genotype-confirmed animals was available from breeding colonies. To minimize confounders, we used color-coded cage cards, unique mouse ID tags, and a two-person verification process for animal identification and treatment preparation. Only the study coordinator and a secondary verifier were aware of group assignments. All personnel performing BLM injections, body-weight monitoring, histology, ELISA, western blotting, and Olink analyses were blinded to group allocation during experimental procedures and data analysis. Data are presented as mean ± standard error of the mean (SEM), with individual values shown. Statistical analyses were performed with GraphPad Prism 10 (GraphPad Software, San Diego, CA). The normality and homoscedasticity of the variables were determined using Shapiro-Wilk and Levene’s tests, respectively. Group comparisons were conducted by one-way or two-way ANOVA with Tukey’s *post hoc* test for multiple comparisons. For comparisons between two independent groups, a one-tailed unpaired Student’s t-test was used, assuming independence between groups.

## Results

### Deficiency of TLR7 or IRF7 attenuates BLM-induced SSc

Given that BLM-induced SSc severity varies by sex, dose, and formulation (31), we conducted pilot studies in both male and female WT mice to identify a BLM formulation and dose that produced a reproducible, non-lethal phenotype characterized by measurable body weight loss and involvement of both skin and lung tissues. Male and female WT mice (10- to 12-week-old) were administered escalating daily subcutaneous doses of either pharmaceutical- or commercial-grade BLM for up to four weeks.

In male mice, pharmaceutical-grade BLM at 5 or 7.5 mg/kg/day caused rapid and progressive weight loss, with 100% of animals reaching humane endpoint for euthanasia by day 10 (BW loss >25%) (**Supplementary Figure 1A**). In contrast, 2 and 2.5 mg/kg/day produced more gradual weight loss by day 28, with the 2.5 mg/kg/day dose inducing greater weight loss than 2 mg/kg/day (15.2% vs 5.9%, p < 0.0001) (**Supplementary Figure 1A**). In female mice, pharmaceutical-grade BLM at 5 mg/kg/day induced greater weight loss than the lower doses, reaching led to 21% by day 28 without animals reaching the humane endpoint (p < 0.0001). Doses of 3, 2.5, and 2 mg/kg/day caused more modest weight losses of 6.6%, 7.4%, and 2.5%, respectively, by day 28 (**Supplementary Figure 1B**).

For commercial-grade BLM, 5 mg/kg/day in male mice caused rapid weight loss and high mortality by day 14, whereas 2.5 mg/kg/day produced measurable but non-lethal weight loss of 18.2% by day 28 (**Supplementary Figure 1C**). In female mice, commercial-grade BLM at 2.5 mg/kg/day caused minimal weight loss (1.8%), while 5 mg/kg/day reduced body weight by 11.3% without reaching humane endpoints (p < 0.0001) (**Supplementary Figure 1D**). A direct comparison of 2.5 mg/kg/day in male mice showed that commercial-grade BLM induced significantly greater weight loss than pharmaceutical-grade BLM between days 4 and 18, without increasing mortality (p < 0.05) (**Supplementary Figure 1E**). Therefore, commercial-grade BLM at 2.5 mg/kg/day was selected for the main study because it generated a measurable, non-lethal phenotype with an appropriate dynamic range for detecting genotype-dependent differences.

We next performed a pilot experiment to test whether TLR7 deficiency attenuated BLM-induced weight loss. WT mice treated with commercial-grade BLM lost approximately 25% of their BW by day 18, whereas BLM-treated *Tlr7^−^/^−^* mice lost approximately 12.5% (p < 0.0001) (**Supplementary Figure 1F**). Together, these findings supported the use of male mice treated with commercial-grade BLM at 2.5 mg/kg/day for 28 days as a sensitive, non-lethal SSc-like model, in which genotype-dependent differences could be detected.

**Figure 1:**
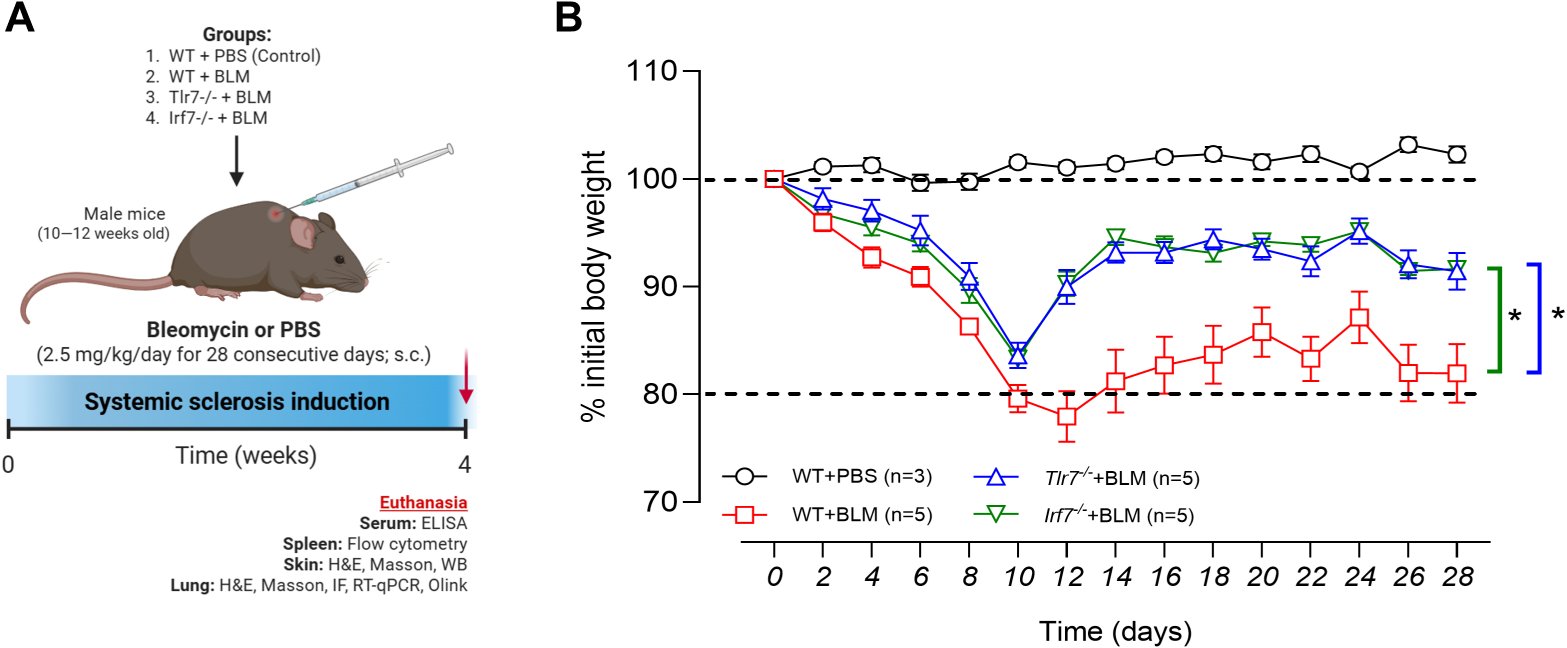
Study design and changes in body weight following bleomycin administration. **(A)** Male mice (10–12 weeks old) were allocated to the following experimental groups: WT+PBS (n=3), WT+BLM (n=5), *Tlr7*^^−^/^−^^+BLM (n=5), and *Irf7*^^−^/^−^^+BLM (n=5). Mice received subcutaneous injections of bleomycin (BLM, 2.5 mg/kg/day, daily) or PBS for 28 consecutive days. After 28 days, animals were euthanized and tissues were collected for downstream analyses: serum for ELISA, spleen for flow cytometry, skin for histology (H&E and Masson’s trichrome) and Western blot, and lung for histology (H&E and Masson’s trichrome), immunofluorescence, RT-qPCR, and Olink proteomics. **(B)** Percentage of body weight loss after BLM treatment. The dashed lines indicate 100% and 80% body weight. BLM-treated *Tlr7^−^/^−^* and *Irf7^−^/^−^* mice showed significantly attenuated weight loss compared with BLM-treated WT mice. Data are presented as mean ± SEM. Statistical analysis was performed using two-way ANOVA followed by Tukey’s *post hoc* test. *p < 0.05*.

Based on the results of this pilot study, we examined the role of TLR7–IRF7 signaling in BLM-induced SSc. Male WT, *Tlr7^−^/^−^*, and *Irf7^−^/^−^* mice received daily subcutaneous BLM injections in the dorsal area for 28 consecutive days, while PBS-treated WT mice served as controls (**Figure 1A**). BLM treatment induced body weight loss in all groups, but *Tlr7^−^/^−^* and *Irf7^−^/^−^* mice showed improved recovery compared with WT mice. By day 28, WT+BLM mice reached 81.9% of baseline BW, whereas *Tlr7^−^/^−^* and *Irf7^−^/^−^* mice maintained 91.4% and 91.6% of baseline BW, respectively (+11.6%, p < 0.05; +11.8%, p < 0.05, respectively) (**Figure 1B**). These data indicate that loss of TLR7 or IRF7 improved systemic tolerance to BLM-induced damage.

**Figure 2:**
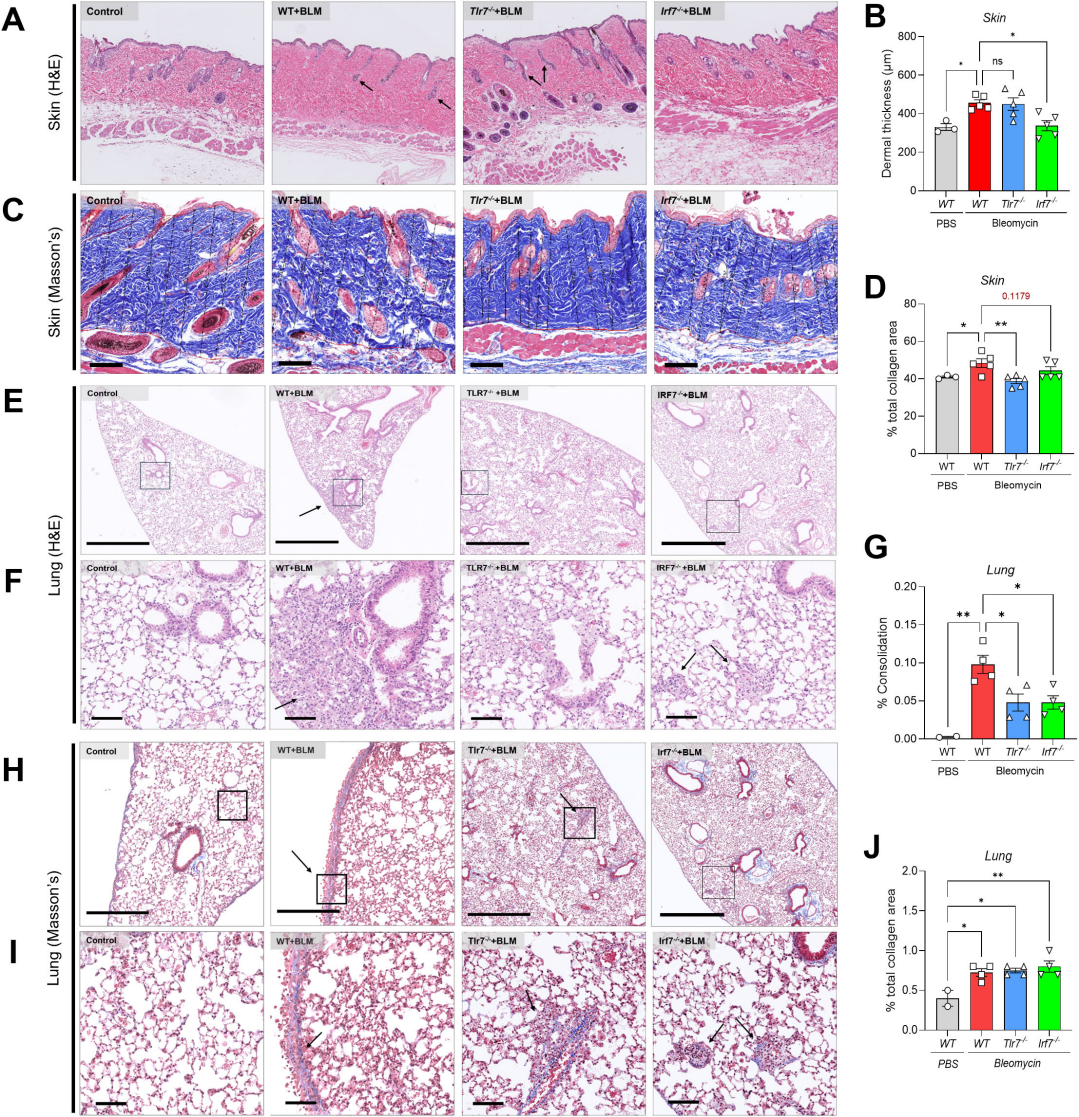
Histological analyses of skin and lung following bleomycin administration. Mice were injected daily with bleomycin (BLM; 2.5 mg/kg) or PBS (Control) for 28 days to induce systemic sclerosis. Skin and lung tissues were collected on day 29 and processed for histopathology. H&E-stained skin sections **(A)** and quantitative analysis of dermal thickness **(B)**. Masson’s trichrome–stained skin sections **(C)** and quantitative analysis of collagen area **(D)**. Representative H&E-stained lung sections at low (scale bar, 1 mm) **(E)** and high (scale bar, 100 μm) magnification **(F)**. Arrows indicate interstitial fibrosis and dense peribronchiolar immune cell infiltration. **(G)** Comparisons of lung inflammation scores. Representative Masson’s trichrome–stained lung sections at low (scale bar, 1 mm) **(H)** and high (scale bar, 100 μm) magnification **(I)**. **(J)** Collagen area quantification. Data are presented as mean ± SEM with individual values. Of note, for skin analyses shown in panels A–D, group sizes were WT+PBS (n=3), WT+BLM (n=5), *Tlr7^−^/^−^*+BLM (n=5), and *Irf7^^−^/^−^^*+BLM (n=5). For lung analyses shown in panels **(E-J)**, group sizes were WT+PBS (n=2), WT+BLM (n=4), *Tlr7^−^/^−^*+BLM (n=4), and *Irf7^^−^/^−^^*+BLM (n=4). Lung sample sizes were smaller because of limited sample availability. Statistical analysis was performed using one-way ANOVA followed by Tukey’s *post hoc* test. *p < 0.05, **p < 0.01; ns, not significant.

We next assessed whether this protection was accompanied by changes in BLM-induced skin pathology. To prevent artifacts from local injections, skin was collected from areas distant from the BLM administration site. BLM-treated mice exhibited key features of SSc, including dermal appendage atrophy and tissue disorganization (**Figures 2A, C**, **Supplementary Figure 2**). Compared with PBS-treated WT mice, WT+BLM mice showed increased dermal thickness (+37.9%, p < 0.05) and collagen deposition (+18.0%, p < 0.05). Dermal thickness was reduced in BLM-treated *Irf7^−^/^−^* mice, but not in *Tlr7^−^/^−^* mice, compared to WT+BLM mice (−25.9%, p < 0.05; −1.3%, p = 0.998, respectively) (**Figures 2A, B**). In contrast, collagen deposition was reduced in *Tlr7^−^/^−^* mice and showed a trend toward reduction in *Irf7^−^/^−^* mice compared to WT+BLM mice (−19.8%, p < 0.01; −8.3%, p = 0.1179, respectively) (**Figures 2C, D**). Together, these findings suggest that TLR7 and IRF7 may contribute to BLM-induced skin pathology through partially distinct mechanisms.

We next examined whether TLR7–IRF7 signaling contributed to BLM-induced lung pathology. BLM-treated WT mice showed marked pulmonary inflammation compared to PBS-treated WT mice (p < 0.01) (**Figures 2E–G**). This response was reduced by approximately half in both *Tlr7^−^/^−^*+BLM and *Irf7^−^/^−^*+BLM mice compared to WT+BLM mice (−51.1%, p < 0.05; −51.0%, p < 0.05, respectively) (**Figures 2E–G**). Collagen deposition was also significantly increased in BLM-treated WT mice compared to PBS-treated WT mice (**Figures 2H–J**). However, no significant differences were observed among BLM-treated WT, *Tlr7^−^/^−^*, and *Irf7^−^/^−^* groups (**Figures 2H–J**). These findings suggest that TLR7 and IRF7 primarily contribute to BLM-induced pulmonary inflammation/consolidation, whereas collagen accumulation in the lung is less affected under these experimental conditions.

To further determine whether the reduction in pulmonary consolidation was accompanied by changes in extracellular matrix remodeling or macrophage-associated markers, we performed lung immunofluorescence for fibronectin, α-SMA, and CD206. Fibronectin staining was similar across groups (**Supplementary Figures 2A, B**). Although α-SMA staining appeared higher in BLM-treated WT mice compared to PBS-treated WT mice, no significant differences were detected among BLM-treated WT, *Tlr7^−^/^−^*, and *Irf7^−^/^−^* mice (**Supplementary Figures 3A, C**). Similarly, the macrophage area and cell size for CD206 also appeared to be increased in BLM-treated WT mice compared to PBS-treated WT mice. Still, no statistically significant differences were detected among BLM-treated WT, *Tlr7^−^/^−^*, and *Irf7^−^/^−^* mice (**Supplementary Figures 4A–C**). Therefore, under these experimental conditions, TLR7 or IRF7 deficiency reduced lung inflammation without significantly affecting extracellular matrix remodeling or macrophage polarization, indicating that TLR7–IRF7 signaling plays a role in the development of pulmonary interstitial pneumonia.

### Deficiency of TLR7 and IRF7 reduced inflammatory monocytes in the spleen

The spleen is a central reservoir and activation site for various immune cells (35). Therefore, we investigated systemic immune dysregulation in the spleens of BLM-induced SSc mice using flow cytometry. BLM-treated WT mice showed a marked increase in inflammatory monocytes compared to PBS-treated WT mice (280.8%, P < 0.001) (**Supplementary Figures 5A, B**). This expansion was reduced in BLM-treated *Tlr7^−^/^−^* and *Irf7^−^/^−^* mice compared to BLM-treated WT mice (-42.1%, p < 0.01; -47.8%, p < 0.01, respectively) (**Supplementary Figures 5A, B**), suggesting that TLR7–IRF7 signaling contributes to inflammatory monocyte accumulation in this model. In contrast, splenic macrophages appeared modestly elevated in BLM-treated WT compared to PBS-treated WT mice, but no statistically significant differences were observed among BLM-treated WT, *Tlr7^−^/^−^, and Irf7^−^/^−^* mice (**Supplementary Figures 5C, D**). Total T cells were reduced in BLM-treated WT compared to PBS-treated WT mice (-29.0%, P < 0.05); however, no differences were observed among BLM-treated WT, *Tlr7^−^/^−^, and Irf7^−^/^−^* mice (**Supplementary Figures 5E, F**). B cell percentages were similar among groups (**Supplementary Figures 5E–G**).

### Deficiency of TLR7 and IRF7 attenuated serum anti-Scl-70 levels

**Figure 3:**
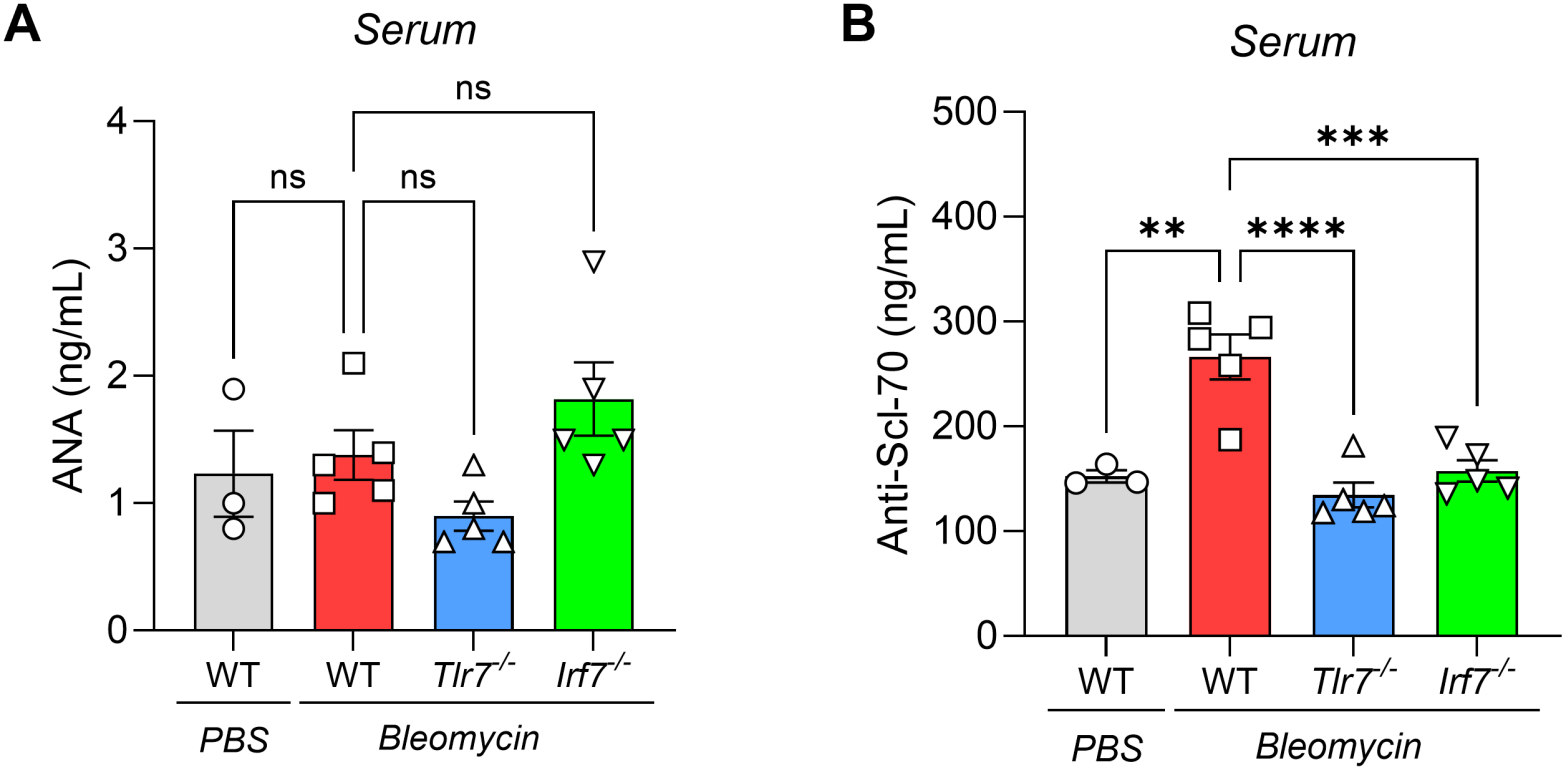
Serum autoantibody levels after bleomycin administration. Mice were injected daily with bleomycin (BLM; 2.5 mg/kg) or PBS (Control) for 28 days to induce experimental systemic sclerosis. After 28 days, blood was collected by cardiac puncture, and serum was isolated by centrifugation. ELISA kits were used to quantify antinuclear antibodies (ANA) and anti-topoisomerase I (anti-Scl-70) levels. **(A)** ANA levels were uniformly low (1–2 ng/mL; dilution 1:10) and did not differ among groups. **(B)** Anti-Scl-70 levels were significantly higher in BLM-treated WT mice compared with PBS-treated WT and BLM-treated *Tlr7^–^/^–^* and *Irf7^–^/^–^* mice. Data are presented as mean ± SEM with individual values. Group sizes for ELISA analyses were WT+PBS (n=3), WT+BLM (n=5), *Tlr7*^^−^/^−^^+BLM (n=5), and *Irf7^^−^/^−^^*+BLM (n=5), based on serum sample availability and assay capacity. Statistical analysis was performed using one-way ANOVA followed by Tukey’s *post hoc* test. **p < 0.01, ***p < 0.001, ****p < 0.0001; ns, not significant.

In SSc patients, antinuclear antibodies (ANA) are commonly detected and serve as a diagnostic hallmark, whereas anti-Scl-70 antibodies are strongly associated with diffuse cutaneous SSc, severe internal organ involvement, and poor prognosis (36). These antibodies exacerbate disease by promoting immune complex formation, complement activation, and tissue injury (37). We therefore measured serum ANA and anti-Scl-70 levels in PBS-treated mice and BLM-treated WT, *Tlr7^−^/^−^* and *Irf7^−^/^−^*. To optimize detection, ANA levels were reassessed using a range of serum dilutions, and the condition yielding the strongest signal was selected for analysis. Despite this, ANA levels remained low across groups (1–2 ng/mL) and did not differ significantly among groups (**Figure 3A**). In contrast, BLM-treated WT mice showed increased serum anti-Scl-70 levels compared to PBS-treated WT mice (+74.9%, p < 0.01). These elevated anti-Scl-70 levels were markedly reduced in BLM-treated *Tlr7^−^/^−^* and *Irf7^−^/^−^* groups compared to WT+BLM group (-49.5%, p < 0.0001; -40.9%, p < 0.001, respectively) (**Figure 3B**). Thus, although ANA remained low under optimized assay conditions, potentially reflecting limited sensitivity rather than absence of autoantibody responses, anti-Scl-70 provided a more robust and disease-relevant serologic readout of autoreactivity in this model.

### Deficiency of TLR7 and IRF7 attenuates pulmonary type I IFN response

**Figure 4:**
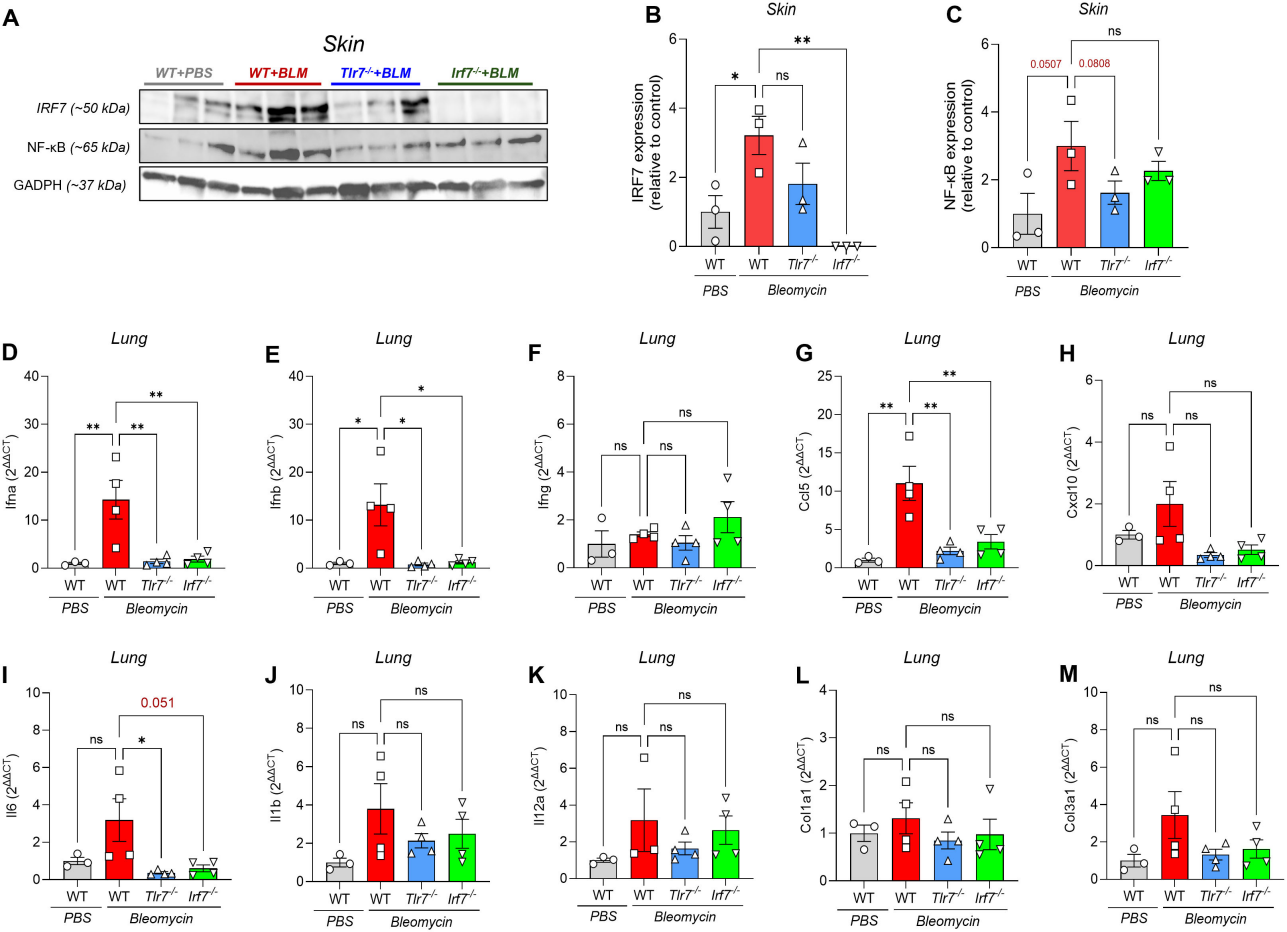
IRF7/NF-κB protein levels in the skin and gene expression in the lung following bleomycin administration. Skins collected from the mice treated with BLM and PBS for 29 days were dissected and processed for Western blot **(A–C)**, and lung fragments were processed for RT-qPCR **(D–M)**. **(A)** Representative blots of IRF7 (~50 kDa), NF-κB p65 (~65 kDa), and GAPDH (~37 kDa, loading control) in skin. **(B)** Densitometric quantification of IRF7. **(C)** Densitometric quantification of NF-κB p65. For Western blot analyses shown in panels A–C, n = 3 mice per group, according to sample availability and gel-loading capacity. **(D–M)** Relative mRNA expression in lung homogenates: **(D)** *Ifna* and **(E)** *Ifnb* in the lungs. **(F)** *Ifng* expression showed no significant differences. **(G)** *Ccl5* was significantly increased in BLM-treated WT mice than in PBS-treated WT and BLM-treated *Tlr7^−^/^−^* and *Irf7^−^/^−^* mice. **(H)** *Cxcl10* expression showed no significant changes. **(I)** *Il6* was upregulated in BLM-treated WT mice compared to BLM-treated *Tlr7^−^/^−^* and *Irf7^−^/^−^* mice. **(J)** *Il1b*, **(K)** *Il12a*, **(L)** *Col1a1*, and **(M)** *Col3a1* expression did not differ among groups. Data are presented as mean ± SEM with individual values. For lung RT-qPCR analyses shown in panels D–M, group sizes were WT+PBS (n=3), WT+BLM (n=4), *Tlr7^^−^/^−^^*+BLM (n=4), and *Irf7^^−^/^−^^*+BLM (n=4). Statistical analysis was performed using one-way ANOVA followed by Tukey’s *post hoc* test. *p < 0.05, **p < 0.01; ns, not significant.

Because TLR7 can signal through both NF-κB-dependent inflammatory pathways and IRF7-dependent type I IFN responses, we first assessed whether these downstream signaling components were altered in BLM-treated mice. IRF7 and total NF-κB p65 protein expression were measured in skin lysates by western blotting (**Figures 4A–C**). BLM-treated WT mice showed increased IRF7 expression compared to PBS-treated WT mice (+221.5%, p < 0.05). IRF7 expression was lower in *Tlr7^−^/^−^*+BLM mice compared to WT+BLM mice, although this difference did not reach statistical significance (−43.6%, p = 0.0808). As expected, IRF7 expression was absent in *Irf7^−^/^−^*+BLM mice (p < 0.01) (**Figure 4B**). Total NF-κB p65 expression showed a trend toward increase in WT+BLM mice compared to PBS-treated WT mice (+200.0%, p = 0.0507), but was not significantly different in *Tlr7^−^/^−^*+BLM or *Irf7^−^/^−^*+BLM mice compared to WT+BLM mice (−45.9%, p = 0.0808; −24.5%, p = 0.1991, respectively) (**Figure 4C**), the small sample size limited statistical power to detect differences. These findings suggest that BLM treatment activates IRF7-associated signaling in skin, whereas changes in total NF-κB p65 were more modest; importantly, NF-κB phosphorylation was not assessed.

We next examined whether these signaling changes were accompanied by altered inflammatory gene expression in the lung, where BLM-induced consolidation was reduced in *Tlr7^−^/^−^* and *Irf7^−^/^−^* mice. Lung RT-qPCR was performed to assess IFNs (*Ifna*, *Ifnb*, *Ifng*), IFN-stimulated genes (*Ccl5*, *Cxcl10*), inflammatory cytokines (*Il6*, *Il1b*, *Il12a*), and remodeling-associated genes (*Col1a1*, *Col3a1*) (**Figures 4D–M**). Compared to PBS-treated WT mice, WT+BLM mice showed increased pulmonary *Ifna* (+1328.0%, p < 0.01) and *Ifnb* (+1221.0%, p < 0.05). Both responses were markedly reduced in *Tlr7^−^/^−^*+BLM and *Irf7^−^/^−^*+BLM mice compared to WT+BLM mice (*Ifna*: −90.1%, p < 0.01; −86.7%, p < 0.01; *Ifnb*: −94.1%, p < 0.05; −90.7%, p < 0.05, respectively) (**Figures 4D, E**). In contrast, *Ifng* expression did not differ significantly among groups (**Figure 4F**). These data suggest that TLR7 and IRF7 deficiency preferentially attenuated the pulmonary type I IFN response rather than broadly suppressing all IFN-related genes.

A similar pattern was observed for selected downstream inflammatory mediators. WT+BLM mice showed increased pulmonary *Ccl5* expression compared to PBS-treated WT mice (+1003.0%, p < 0.01). This increase was reduced in *Tlr7^−^/^−^*+BLM and *Irf7^−^/^−^*+BLM mice compared to WT+BLM (−79.9%, p < 0.01; −69.1%, p < 0.01, respectively) (**Figure 4G**). By contrast, *Cxcl10* expression showed no significant differences among groups (**Figure 4H**). Pulmonary *Il6* expression was higher in WT+BLM mice than in PBS-treated WT mice (+218.8%, p = 0.051) (**Figure 4I**). *Il6* expression was reduced in *Tlr7^−^/^−^*+BLM mice and showed a similar trend in *Irf7^−^/^−^*+BLM mice compared to WT+BLM mice (−88.5%, p < 0.05; −81.0%, p = 0.051, respectively) (**Figure 4I**). In contrast, *Il1b* and *Il12a* did not differ significantly among BLM-treated groups (**Figures 4J, K**). *Col1a1* and *Col3a1* genes were also not significantly altered among BLM-treated groups (**Figures 4L, M**). Overall, these findings support a model in which TLR7–IRF7 signaling preferentially amplifies pulmonary type I IFN, *Ccl5*, and *Il6*-associated inflammatory responses in BLM-induced SSc-like disease, while broader cytokine and remodeling gene changes were less consistently affected.

### TLR7–IRF7 signaling is associated with increased pulmonary CCL2/CCL12 expression in BLM-induced SSc mice

**Figure 5:**
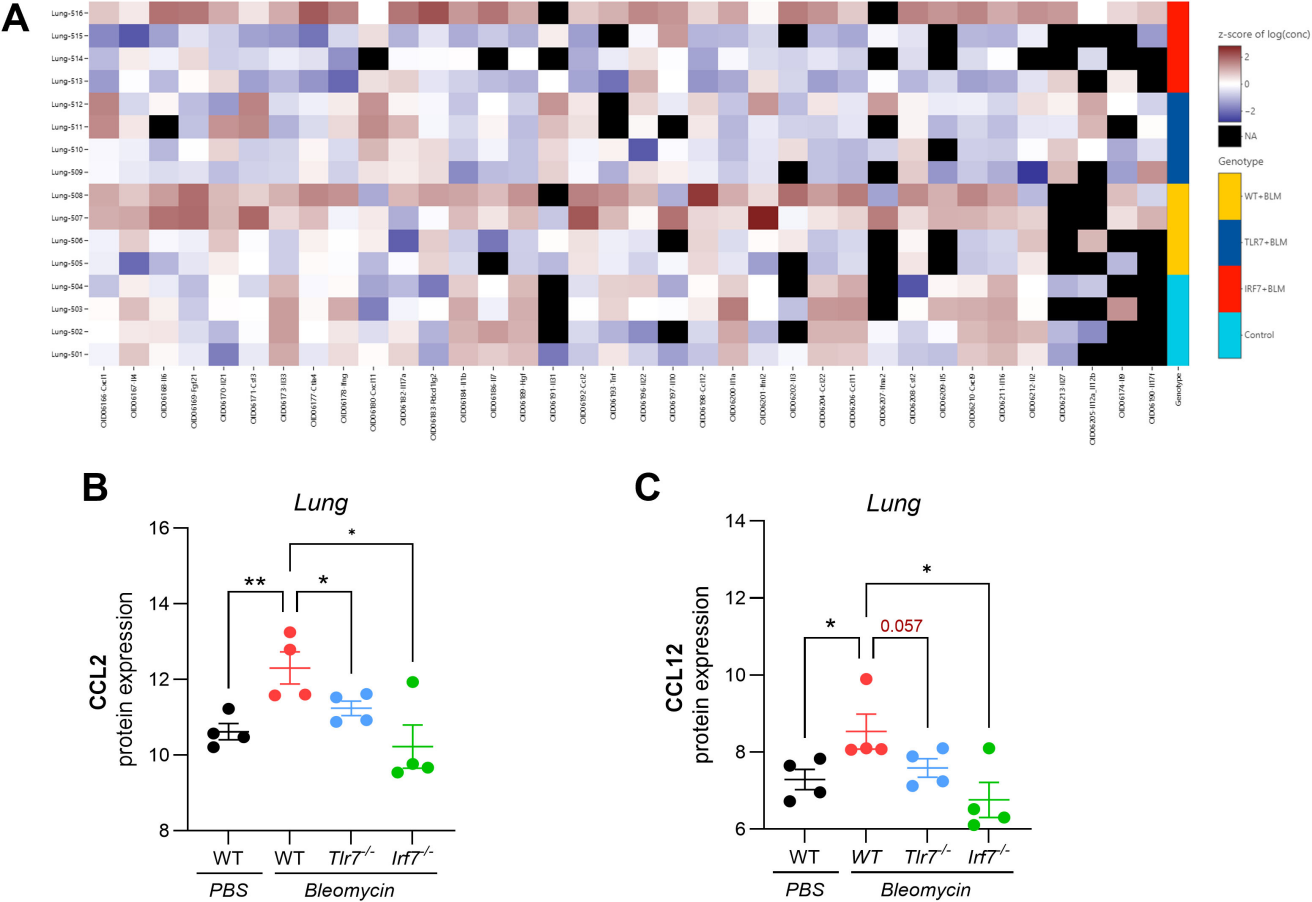
Lung protein expression profile after bleomycin administration. Lungs collected from the mice treated with BLM and PBS for 29 days were dissected, proteins were extracted. The cytokine/chemokine levels were quantified in the homogenates using Olink proteomics platform. **(A)** Heatmap of differentially expressed proteins in lung homogenates. **(B)** Quantification of CCL2 expression: significantly lower expression in BLM-treated *Tlr7^–^/^–^* and *Irf7^–^/^–^* mice compared with BLM-treated WT mice. **(C)** Quantification of CCL12 expression: significantly reduced in PBS-treated WT mice and BLM-treated *Irf7^–^/^–^* mice, with a trend toward reduction in BLM-treated *Tlr7^–^/^–^* mice compared to BLM-treated WT mice. Data are presented as mean ± SEM with individual values. Group size was n = 4 mice per group, based on sample availability and assay capacity. Statistical analysis was performed using one-tailed Student’s t-test. *p < 0.05, **p < 0.01.

We further profiled 44 cytokines and chemokines in mouse lung lysates using the Olink proximity extension assay (**Figure 5A**). Among the analytes examined, CCL2 and CCL12 showed a pattern consistent with the inflammatory lung phenotype observed in WT+BLM mice. Compared to PBS-treated WT mice, WT+BLM mice showed increased pulmonary CCL2 protein expression (+15.9%, p < 0.01) (**Figure 5B**). CCL2 levels were reduced in *Tlr7^−^/^−^*+BLM and *Irf7^−^/^−^*+BLM mice compared to WT+BLM mice (−8.7%, p < 0.05; −16.9%, p < 0.05, respectively) (**Figure 5B**). Similarly, WT+BLM mice showed increased pulmonary CCL12 expression compared to PBS-treated WT mice (+17.1%, p < 0.05) (**Figure 5C**). CCL12 levels showed a reduction in *Tlr7^−^/^−^*+BLM and *Irf7^−^/^−^*+BLM mice compared to WT+BLM mice (−11.1%, p = 0.057; −20.8%, p < 0.05, respectively) (**Figure 5C**). These findings suggest that BLM-induced SSc-like lung inflammation is accompanied by increased pulmonary CCL2/CCL12 expression, which appears to be attenuated by loss of TLR7 and IRF7.

### Inhibition of CCL2/CCL12 activities with their antagonist protects against BLM-induced SSc

**Figure 6:**
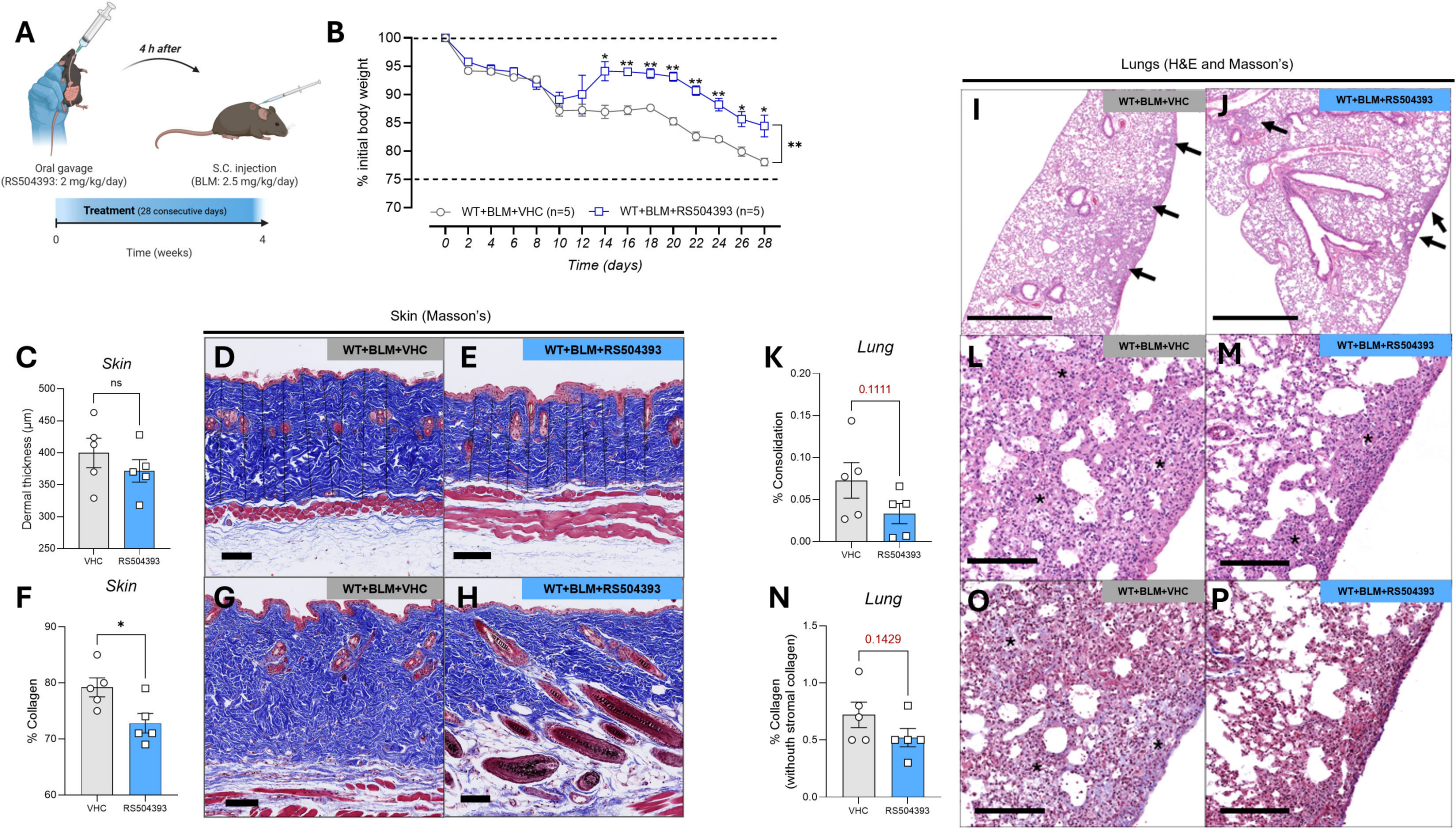
RS504393 treatment. **(A)** Male B6 mice (10–12 weeks old) received RS504393 (2 mg/kg) by oral gavage four hours before the daily subcutaneous BLM injections (2.5 mg/kg) for 28 consecutive days. B6 control mice received vehicle (VHC). **(B)** Percentage of body weight loss after BLM treatment. The dashed line indicates 100% and 75% body weight. **(C)** Dermal thickness between untreated **(D)** and treated mice **(E)**. Bar = 100 μm. **(F)** Quantitative analysis of collagen areas (blue) stained by Masson’s trichrome within the dermis in untreated **(G)** and treated mice **(H)**. Bar = 100 μm. H&E staining of pulmonary consolidations (arrows) in untreated **(I)** and treated **(J)** mice with a trend towards a lower proportion of the lung being affected in treated mice **(K)**. Bar = 1 mm. Consolidated regions of untreated **(L)** and treated **(M)** mice are characterized by expansion of the pulmonary interstitium by fibrosis (asterisks) and mixed inflammatory infiltrate. Bar = 100 μm. Collagen deposition **(N)** (blue, asterisks) in untreated **(O)** and treated mice **(P)**. Bar = 100 μm. Data are presented as mean ± SEM with individual values (n = 5 mice per group). *p < 0.05, **p < 0.01 were analyzed using a two-way ANOVA followed by Tukey’s *post hoc* test (body weight changes) and one-tailed Student’s t-test (pathology variables).

To test whether pharmacologic inhibition of the downstream CCL2/CCL12-CCR2 axis could attenuate disease features in this model, we treated WT mice with the CCR2 antagonist RS504393. RS504393 was administered by oral gavage at 2 mg/kg/day for 28 consecutive days, with each dose given 4 hours before the daily BLM injection to support systemic CCR2 inhibition during ongoing BLM exposure (34, 38) (**Figure 6A**).

Compared with vehicle-treated mice, RS504393-treated mice showed improved BW maintenance during BLM exposure. By day 28, vehicle-treated mice reached 78.1% of baseline BW, whereas RS504393-treated mice maintained 84.4% of baseline BW (+8.2%, p < 0.01) (**Figure 6B**). In the skin, RS504393 treatment did not significantly alter dermal thickness compared to vehicle-treated control (**Figures 6C–E**). However, Masson’s trichrome staining showed reduced dermal collagen deposition in RS504393-treated mice compared to vehicle-treated mice (−8.1%, p < 0.05) (**Figures 6F–H**).

In the lung, both vehicle- and RS504393-treated mice developed multifocal consolidation after BLM treatment. RS504393-treated mice showed a lower mean proportion of consolidated lung area than vehicle-treated mice, although this difference did not reach statistical significance (−54.3%, p = 0.1111) (**Figures 6I–K**). Similarly, lung collagen accumulation showed a non-significant reduction in RS504393-treated mice compared to vehicle-treated mice (−27.8%, p = 0.1429) (**Figures 6N–P**). Together, these findings suggest that CCR2 antagonism partially attenuates BLM-induced disease features, with clearer effects on BW maintenance and dermal collagen deposition than on lung consolidation or collagen accumulation under these experimental conditions.

## Discussion

The main finding of this study is that TLR7 and IRF7 deficiency attenuated BLM-induced pulmonary inflammation, reduced IFN-I gene expression, decreased serum anti-Scl-70 levels, and reduced pulmonary CCL2/CCL12 expression in male mice. Given that SSc is an autoimmune disease characterized by aberrant activation of innate and adaptive immunity, autoantibody production, and progressive pulmonary impairment, we hypothesized that the TLR7-IRF7 axis contributes to the inflammatory and autoimmune features of BLM-induced SSc-like disease. Consistent with this hypothesis, genetic deficiency in TLR7 and IRF7 was associated with partial protection against systemic disease manifestations, and this effect was attenuated by pharmacological CCR2 antagonism. Collectively, these data corroborate the role of the TLR7-IRF7 axis in modulating immune responses linked to lung injury and autoantibody-associated disease features in this model.

We found that dermal thickening, a hallmark of SSc (1–3) was attenuated in *Irf7^−^/^−^* mice but not in *Tlr7^−^/^−^*. This finding aligns with previous research showing that IRF7 deficiency reduces BLM-induced dermal fibrosis in mice (28), supporting its role in skin fibrosis (9). Previously published results regarding TLR7 deficiency remain inconsistent: while one study reported no protection against dermal fibrosis (39), another showed attenuation of BLM-induced dermal thickening (32). In our study, we detected only lower collagen deposition, not dermal thickness, in BLM-treated *Tlr7^-/-^* mice compared with BLM-treated WT mice. The role of TLR7 in BLM-induced skin fibrosis requires further investigation with larger sample sizes to detect changes.

TLR7 and IRF7 deficiency markedly reduced pulmonary inflammation in our model. Pulmonary involvement is clinically significant in SSc, as ILD is the leading cause of death, with nonspecific interstitial pneumonia affecting up to 65% of patients (3, 40, 41). The reduced pulmonary inflammation in *Tlr7*- and *Irf7*-deficient mice may be associated with the lower numbers of inflammatory monocytes observed in the spleen, consistent with prior research showing that the spleen serves as a reservoir of classical monocytes that can be mobilized to the lungs during inflammatory conditions (42). In addition, monocyte-derived alveolar macrophages contribute to the pathogenesis of SSc-associated interstitial lung disease in human patients (43). However, monocyte infiltration and alveolar macrophages were not directly assessed in the lungs and skin, which warrants further study.

In line with this, flow cytometric profiling of splenic immune populations revealed coordinated changes, including increased inflammatory monocytes, and stable B cell frequencies. Although B cells are the primary source of autoantibodies, CD4+ T cells provide critical regulatory support for B cell activation and differentiation. However, we detected reduced CD4+ T cells in BLM-treated WT compared to PBS-treated WT mice. The apparent dissociation between reduced CD4+ T cells and increased anti-Scl-70 levels likely reflects the combinatorial responses to the BLM challenge (direct BLM cell-damage effects vs. stimulation of the immune response), examined at the terminal time point (28 days). Earlier T cell activation may be sufficient to prime autoreactive B cells, a possibility that requires further investigation.

Because these analyses were performed in the spleen, they may not fully reflect immune dynamics in target tissues such as the lung or skin, which is a limitation of this study. Together, these findings suggest that the TLR7–IRF7 axis promotes systemic mobilization of proinflammatory cells, thereby worsening SSc-ILD. Our findings align with clinical evidence that gain-of-function mutations in TLR7 are linked to severe autoimmune diseases (15, 44). Similarly, mutations in IRF7 increase the risk of systemic lupus erythematosus by transcriptionally regulating IFN-I (45). Although inflammation was reduced in BLM-treated *Tlr7^−^/^−^* and *Irf7^−^/^−^* mice, collagen levels remained high across all BLM-treated groups, suggesting that a four-week treatment period may be too short to detect genotype-specific differences in established fibrosis. To our knowledge, this is the first study to investigate TLR7 and its effector IRF7 in a rodent model of SSc and to explore their roles in lung pathology.

TLR7–IRF7 signaling is a key regulator of IFN-I production, driving both IFN-α and IFN-β responses (9, 46). Sustained activation of this pathway in plasmacytoid dendritic cells (pDCs) has been implicated in autoimmune diseases, where excessive IFN-I release amplifies inflammation and autoreactivity (47). In SSc patients, this persistent hyperactivation generates the so-called “IFN signature” characterized by overexpression of ISGs, which has been associated with worse lung function and is particularly prevalent in patients with diffuse cutaneous disease and ILD (48, 49). BLM-treated WT mice in our study showed increased IFN-I-related gene expression, greater lung inflammation, and higher serum anti-Scl-70 levels. While these findings do not establish direct causality, they suggest that TLR7-IRF7-driven IFN-I responses may amplify inflammatory and autoreactive pathways in this model. Our results support the role of this pathway in pathogenic immune activation. Supporting this, a phase IIb clinical trial found that blocking the IFN-α pathway with sifalimumab improved clinical outcomes in moderate-to-severe SLE, underscoring the relevance of targeting IFN-I signaling (50).

Autoantibodies are key biomarkers in SSc, aiding diagnosis, classification, and prognosis (36). ANA are found in over 90% of SSc patients, often preceding symptoms and predicting microvascular damage and progression from Raynaud’s phenomenon. ANA-negative cases are uncommon yet distinct, highlighting the significance of ANA while underscoring the need for additional markers (36, 51). In our model, serum ANA levels remained low and should be interpreted cautiously. Rather than concluding that autoreactivity was absent, this result may reflect assay sensitivity or the particular autoantibody profile generated in this model. Among SSc-specific autoantibodies, anti-Scl-70 is highly specific and clinically significant, as it identifies patients at risk for diffuse cutaneous involvement, early vasculopathy, and severe ILD (36). Notably, SSc patients positive for anti-Scl-70 have a significantly increased risk of developing ILD and a poorer overall prognosis, a pattern that aligns with our findings (52). In our study, BLM-treated WT mice exhibited higher anti-Scl-70 levels than BLM-treated *Tlr7^−^/^−^* and *Irf7^−^/^−^* mice, supporting the relevance of this autoantibody-associated phenotype in our model. One possible explanation is that BLM-induced tissue injury promotes the release of self-nucleic acids, which may enhance innate immune sensing and favor autoreactive B-cell responses. This mechanism has been described in other autoimmune settings and is consistent with, but not directly proven by, our data (53, 54). This process may stimulate pDCs to promote the proliferation of autoreactive B cells and the production of autoantibodies (55). Overall, our findings support a model in which tissue injury, nucleic acid sensing, IFN-I induction, and autoreactive humoral responses reinforce one another, though the precise cellular sequence remains unclear.

Our findings also identify the CCL2/CCL12-CCR2 axis as a plausible mediator of pulmonary inflammation in this model. Given that CCL2/CCL12 regulates the CCR2-dependent recruitment of inflammatory monocytes and monocyte-derived macrophages, the reduced pulmonary expression of these chemokines in BLM-treated *Tlr7^−^/^−^* and *Irf7^−^/^−^* mice suggests a potential mechanistic link between attenuated innate immune activation and reduced pulmonary inflammation. However, monocyte recruitment to target tissues was not directly assessed in this study. Previous studies show that CCR2 deficiency promotes M2-like alveolar macrophage proliferation and accelerates the resolution of lung injury (56), while pharmacologic CCR2 blockade attenuates lung fibrosis in mice (26). RS504393, a CCR2 antagonist, has been shown to reduce dermal and pulmonary fibrosis when administered locally at the site of injury in the BLM-induced scleroderma mouse model (38). In contrast, we delivered BLM subcutaneously to induce a lung SSc-like phenotype and administered RS504393 orally to evaluate systemic CCR2 inhibition after disease onset. This approach enabled a comprehensive assessment of how CCR2-dependent leukocyte trafficking could modify downstream disease manifestations. Because the CCL2/CCL12–CCR2 axis primarily regulates circulating monocyte mobilization and tissue recruitment, systemic delivery was considered more appropriate to interrogate its role in lung inflammation. Consistent with this rationale, treatment with RS504393 attenuated BLM-induced body weight loss and lung injury, partially recapitulating the protective phenotype observed in *Tlr7^−^/^−^* and *Irf7^−^/^−^* mice. In SSc patients, elevated circulating CCL2 levels predict faster pulmonary decline and worse survival (57). Together with the reduced CCL2 and CCL12 expression in *Tlr7^−^/^−^* and *Irf7^−^/^−^* mice, these findings support further investigation of the CCL2/CCL12–CCR2 axis as a potential therapeutic target in SSc-related inflammatory lung disease.

Several features of the BLM model are critical for interpreting our results, as route, dose, and duration strongly influence the pattern and severity of tissue injury. In our study, the selected subcutaneous low-dose regimen over four weeks induced robust pulmonary inflammation but limited fibrosis, consistent with previous reports (31). This design was intentionally chosen to provide a non-lethal and sensitive framework for detecting genotype-dependent differences, rather than to maximize fibrotic outcomes. It is well recognized that no single animal model fully recapitulates the complexity of SSc, and that each experimental approach captures only selected aspects of the disease (58). While intraperitoneal administration has been successfully used to induce pulmonary fibrosis (59), it may be associated with increased systemic toxicity and higher mortality depending on dose and experimental conditions. Subcutaneous delivery, in contrast, is widely used and enables assessment of both local and distal tissue responses within a single experimental framework.

This study has some limitations. Modest and variable group sizes, due to colony availability and limited tissue yield, may have reduced statistical power. The 4-week SSc induction period likely favored inflammatory lung changes over the development of established fibrosis. While we found associations between TLR7–IRF7 signaling, IFN-I responses, CCL2/CCL12 expression, and pulmonary inflammation, the precise cellular sources and causal sequence remain unclear. Future research using lineage-specific and cell-resolved approaches is needed to clarify these mechanisms. In addition, although SSc is more prevalent in women, the main study was performed in male mice to enable clearer detection of genotype-dependent differences under the selected BLM conditions. This represents a limitation, as potential sex-dependent differences in disease progression were not assessed and should be addressed in future studies. Finally, the BLM-induced SSc model only partially reflects the complexity, chronicity, and sex distribution of human disease, thereby limiting the direct translation of our findings.

In conclusion, our findings support a model in which TLR7–IRF7-dependent IFN-I signaling contributes to pulmonary inflammation and autoantibody-associated disease features in BLM-induced SSc-like disease, at least in part through downstream engagement of the CCL2/CCL12–CCR2 axis. These results refine the link between innate immune sensing and lung inflammation in this model and support further investigation of downstream chemokine pathways as potential therapeutic targets in SSc.

## Data Availability

The original contributions presented in the study are included in the article/**Supplementary Material**, further inquiries can be directed to the corresponding author/s.
