# Supplementary material for: TLR7 signaling aggravates lung inflammation associated with increased anti-Scl-70 autoantibody production in murine bleomycin-induced systemic sclerosis
Source: Front Immunol. 2026 Jul 1;17:1823229. doi: 10.3389/fimmu.2026.1823229 (PMC13368542; doi:10.3389/fimmu.2026.1823229)
Supplement: Supplementary file 1 [file DataSheet1.docx]

**TLR7 signaling aggravates lung inflammation associated with increased anti-Scl-70 autoantibody production in murine bleomycin-induced systemic sclerosis**

J.F. Evangelista, A.K.N. Vidal, D. Xu, M. Islamuddin, Chen. Y, Wang. C, R. Freitas, S. Liu, E. Engler-Chiurazzi, R. V. Blair, P. Datta, X. Qin

Correspondence to: [xqin2@tulane.edu](mailto:xqin2@tulane.edu)

**Supplementary Materials and Methods**

**Supplementary Tables**

**Table S1.** Antibodies for Immunofluorescence.

**Table S2.** Antibodies for Immunoblotting.

**Table S3.** Primer sequences used for RT-qPCR.

**Supplementary Figures**

**Supplementary Figure 1.** Histological analysis.

**Supplementary Figure 2.** Pilot study to determine the optimal dose and formulation of bleomycin (BLM) for the induction of weight loss in WT mice.

**Supplementary Figure 3.** Lung immunofluorescence for fibronectin and α-SMA after bleomycin administration.

**Supplementary Figure 4.** Lung immunofluorescence for CD206 and quantification of macrophage size after bleomycin administration.

**Supplementary Figure 5. Immune cell composition in the spleen after bleomycin administration.**

**Supplementary materials and methods:**

**Flow cytometry.**

Single-cell suspensions were prepared from mouse spleens by mechanical dissociation through a 40 μm nylon cell strainer, followed by ACK lysis of red blood cells. Cells were resuspended in FACS buffer (PBS + 2% fetal bovine serum) and incubated with anti-CD16/32 (1:200; clone 93, eBioscience, Cat# 48-0161-80) to block Fc receptors. Viability was assessed using Aqua LIVE/DEAD dye (Invitrogen, Cat# L34957A). For surface staining, cells were incubated with the following fluorochrome-conjugated antibodies: CD45-eFluor450 (clone 30-F11, eBioscience, Cat# 48-0451-82), CD11b-PE-Cy7 (clone M1/70, Invitrogen, Cat# 25-0112-82), Ly-6C-FITC (clone HK1.4, BioLegend, Cat# 128006), F4/80-BV605 (clone BM8, BioLegend, Cat# 123133), CD3-APC (clone 17A2, Invitrogen, Cat# 17-0032-82), and CD19-PE (clone 1D3, Invitrogen, Cat# 12-0193-82). Samples were acquired on a BD LSRFortessa and analyzed using FACSDiva v.6.1.3 software (BD Biosciences).

**Enzyme-linked immunosorbent assay (ELISA).**

Serum levels of ANA (Creative Diagnostics, DEIA-BJ2332) and anti-Scl-70 (Creative Diagnostics, DEIA117J) were measured using a commercial ELISA kit according to the manufacturer’s instructions.

**Histological analysis.**

Fixed lung and skin tissues were paraffin-embedded, sectioned, deparaffinized, and stained with hematoxylin and eosin or Masson's trichrome **(24)**.

**Immunofluorescence.**

Paraffin-embedded lung sections (5 µm) were deparaffinized, rehydrated, and subjected to antigen retrieval (citrate buffer, pH 6.0, or Tris-EDTA buffer, pH 9.0, 95°C for 10 min). After blocking with 5% BSA for one hour at room temperature, sections were incubated overnight at 4°C with primary antibodies **(Supplementary Table S1)**. Images were acquired with a confocal microscope. Quantification was performed using HALO AI 3.4 software (Indica Labs).

**Immunoblotting.**

Skin lysates were quantified for protein concentration using the BCA assay (Thermo Fisher Scientific). Samples were separated on 10% SDS-PAGE gels and transferred to nitrocellulose membranes. Membranes were blocked with EveryBlot blocking buffer (Bio-Rad) and incubated with primary **(Supplementary Table S2)**. Immunoreactive bands were visualized using enhanced chemiluminescence (Santa Cruz Biotechnology) on a ChemiDoc™ MP Imaging System (Bio-Rad). Band intensities were quantified with ImageJ software and expressed as arbitrary units.

**Quantitative reverse transcription polymerase chain reaction (qRT-PCR).**

Total RNA was extracted from frozen lung tissue using the RNeasy Mini Kit (Qiagen, Cat# 74104). RNA concentration was determined with the NanoDrop 2000 spectrophotometer (Thermo Fisher Scientific). cDNA synthesis was performed with the High-Capacity cDNA Reverse Transcription Kit (Invitrogen). qPCR was run on a StepOne Plus Real-Time PCR System (Applied Biosystems) with SYBR Green Master Mix (Qiagen). Primer sequences are listed in **Supplementary Table S3**. β-actin was used as housekeeping control. Fold changes were calculated using the 2*^-ΔΔ^*^Ct^ method.

**Olink proteomics.**

Lung fragments were homogenized in RIPA buffer and analyzed with the Olink^®^ Target 48 Cytokine panel (Olink Proteomics AB, Thermo Fisher Scientific), based on the Proximity Extension Assay technology, according to the manufacturer’s instructions. Data were normalized and expressed as Normalized Protein eXpression (NPX). Heatmaps were generated using NPX values via the Olink analysis tool ([www.olink.com/resources-support/analysis-tools/](http://www.olink.com/resources-support/analysis-tools/)).

**Table S1.** Antibodies for Immunofluorescence.

| **Target** | **Antibody Information** |
| --- | --- |
| α-SMA | AF647; Abcam, Cat# AB202296 |
| Fibronectin | AF488; BD Pharmingen, Cat# 563100 |
| CD206 | PE; Biolegend, Cat# 141705 |
| DAPI | Invitrogen, Cat# D1306 |

**Table S2.** Antibodies for Immunoblotting.

| **Target** | **Antibody Information** |
| --- | --- |
| IRF7 | 45–60 kDa, rabbit, 1:500, #72073, Cell Signaling |
| NF-κB p65 | 65 kDa, rabbit, 1:1000, #8242, Cell Signaling |
| GAPDH | 37 kDa, rabbit, 1:10000, #2118, Cell Signaling |

**Table S3.** Primer sequences used for RT-qPCR.

| **Target gene** | **Primer** | **Nucleotide sequence (5’-3’)** |
| --- | --- | --- |
| *Ifna* | Forward | TGCCCAGCAGATCAAGAAGG |
|  | Reverse | TCAGGGGAAATTCCTGCACC |
| *Ifnb* | Forward | GTACAACAGCTACGCCTGGA |
|  | Reverse | GAGTCCGCCTCTGATGCTTA |
| *Ifng* | Forward | AAAGAGATAATCTGGCTCTGC |
|  | Reverse | GCTCTGAGACAATGAACGCT |
| *Ccl5* | Forward | GCCCACGTCAAGGAGTATTTCTA |
|  | Reverse | TCGTGGCAATGATCTCAACAC |
| *Cxcl10* | Forward | GCCCACGTCAAGGAGTATTTCTA |
|  | Reverse | ACACACTTGGCGGTTCCTTC |
| *Il6* | Forward | TAGTCCTTCCTACCCCAATTTCC |
|  | Reverse | TTGGTCCTTAGCCACTCCTTC |
| *Il1b* | Forward | GCAACTGTTCCTGAACTCAACT |
|  | Reverse | ATCTTTTGGGGTCCGTCAACT |
| *Il12a* | Forward | CAGCATGTGTCAATCACGCTAC |
|  | Reverse | TGTGGTCTTCAGCAGGTTTC |
| *Col1a1* | Forward | CGCCATCAAGGTCTACTGC |
|  | Reverse | ACGGGAATCCATCGGTCA |
| *Col3a1* | Forward | GCCCACAGCCTTCTACACCT |
|  | Reverse | GCCAGGGTCACCATTTCTC |
| *β-actin* | Forward | GCTCCTAGCACCATGAAGAT |
|  | Reverse | GTGTAAAACGCAGCTCAGTA |

**
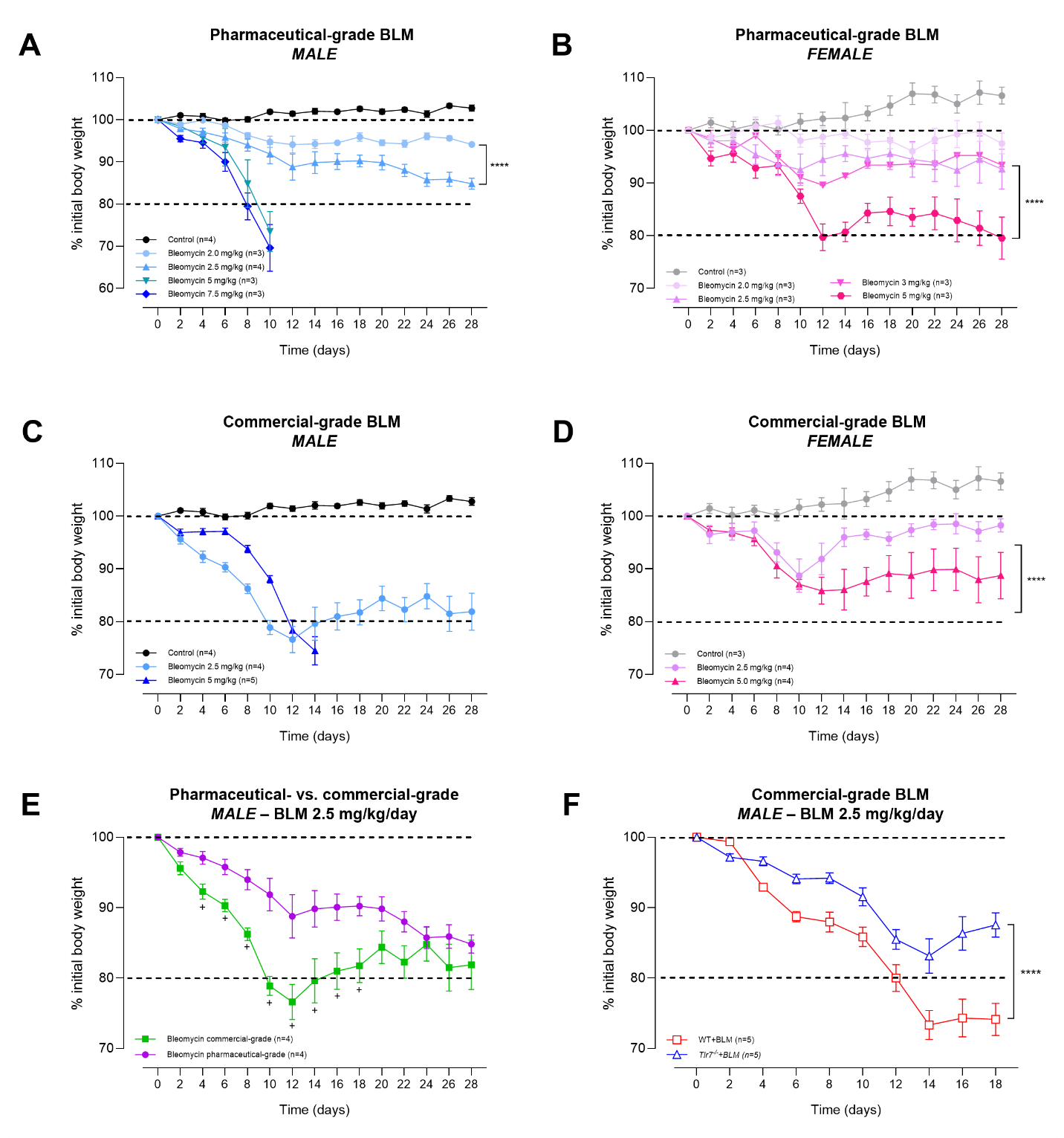
**

**Supplementary Figure 1. Pilot study to determine the optimal dose and formulation of bleomycin (BLM) for weight loss induction in WT mice.** Male and female mice were injected daily with bleomycin (BLM; 2–7.5 mg/kg) or PBS (Control) for up to 28 days to induce experimental systemic sclerosis. Body weight was recorded every other day**. (A)** Male WT mice treated with pharmaceutical-grade BLM (2, 2.5, 5, or 7.5 mg/kg): higher doses (5 and 7.5 mg/kg) caused rapid body weight loss and high mortality, whereas lower doses (2 and 2.5 mg/kg) induced progressive weight reduction maintained until day 28. **(B)** Female WT mice treated with pharmaceutical-grade BLM (2, 2.5, 3, or 5 mg/kg): The 5 mg/kg dose resulted in marked and sustained weight loss compared to the lower doses. **(C)** Male WT mice treated with commercial-grade BLM (2.5 or 5 mg/kg): the 5 mg/kg dose led to high mortality, while the 2.5 mg/kg dose caused ~20% weight loss by day 28. **(D)** Female WT mice treated with commercial-grade BLM (2.5 or 5 mg/kg): The 5 mg/kg dose resulted in a ~10% reduction in body weight compared with the 2.5 mg/kg dose. **(E)** Direct comparison of pharmaceutical- vs. commercial-grade BLM at 2.5 mg/kg in male WT mice: commercial-grade BLM induced significantly greater weight loss between days 4 and 18. **(F)** Pilot experiment with commercial-grade BLM (2.5 mg/kg/day) in male WT and *Tlr7^–^/^–^* mice for 18 days: *Tlr7^–^/^–^* mice exhibited significantly attenuated weight loss compared with WT mice. Data are presented as mean ± SEM. Statistical analysis was performed using two-way ANOVA followed by Tukey’s post hoc test. **p* < 0.05, *****p* < 0.0001.

**Supplementary Figure 2. Histological analysis.** Sections (5 µm) were scanned using the Axio Scan.Z1 (Zeiss, Thornwood, NY), and images were analyzed with HALO Figure Maker software (Indica Labs, Albuquerque, NM). Dermal thickness was determined by repetitive measurements from the base of the epidermis to the base of the dermis in regions with perpendicular orientation. Here we present a representative image from quantitative analysis of collagen deposition in skin. Untreated mouse. **A)** Increased dermal collagen deposition in a mouse receiving bleomycin. **B)** The analysis masks a deep learning algorithm to recognize dermal collagen (cyan). Masson’s Trichrome. Bar =500 um. Lung inflammation was scored using the Ashcroft scale across the entire lung area with an AI-assisted tool (HALO), validated by an experienced pathologist who was blinded to the group allocation. Collagen deposition was quantified as the percentage of stained areas relative to total tissue area.

**
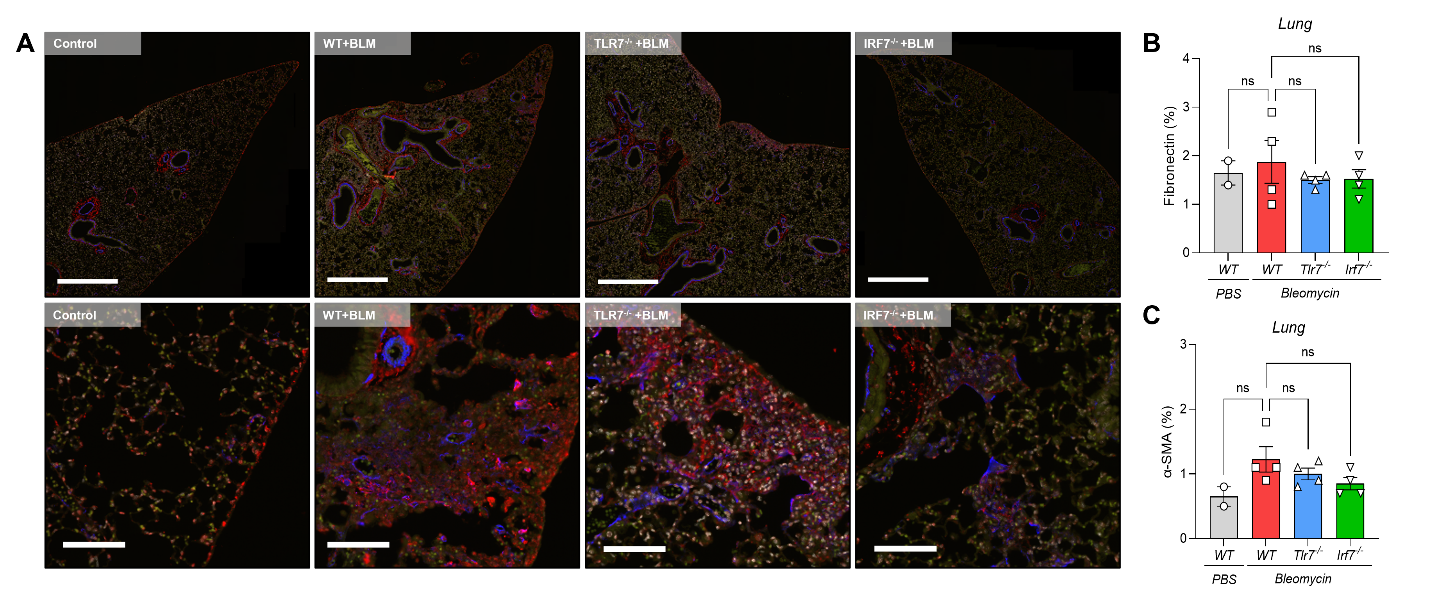
**

**Supplementary Figure 3. Lung immunofluorescence for fibronectin and α-SMA after bleomycin administration.** Mice were injected daily with bleomycin (BLM; 2.5 mg/kg) or PBS (Control) for 28 days to induce experimental systemic sclerosis. Lungs were collected on day 29 and processed for immunofluorescence staining. **(A)** Representative images of lung sections stained for fibronectin (red), α-smooth muscle actin (α-SMA; blue), and nuclei (white). Upper panel: low magnification (scale bar: 500 μm). Lower panel: high magnification (scale bar: 100 μm). **(B)** Quantification of fibronectin (% area): no significant differences among groups. **(C)** Quantification of α-SMA (% area): no significant differences among groups. Data are presented as mean ± SEM with individual values. Group sizes: WT+PBS (n=2), WT+BLM (n=4), *Tlr7*^⁻/⁻^+BLM (n=4), and *Irf7*^⁻/⁻^+BLM (n=4). Statistical analysis was performed using one-way ANOVA followed by Tukey’s post hoc test. ns, not significant.

**
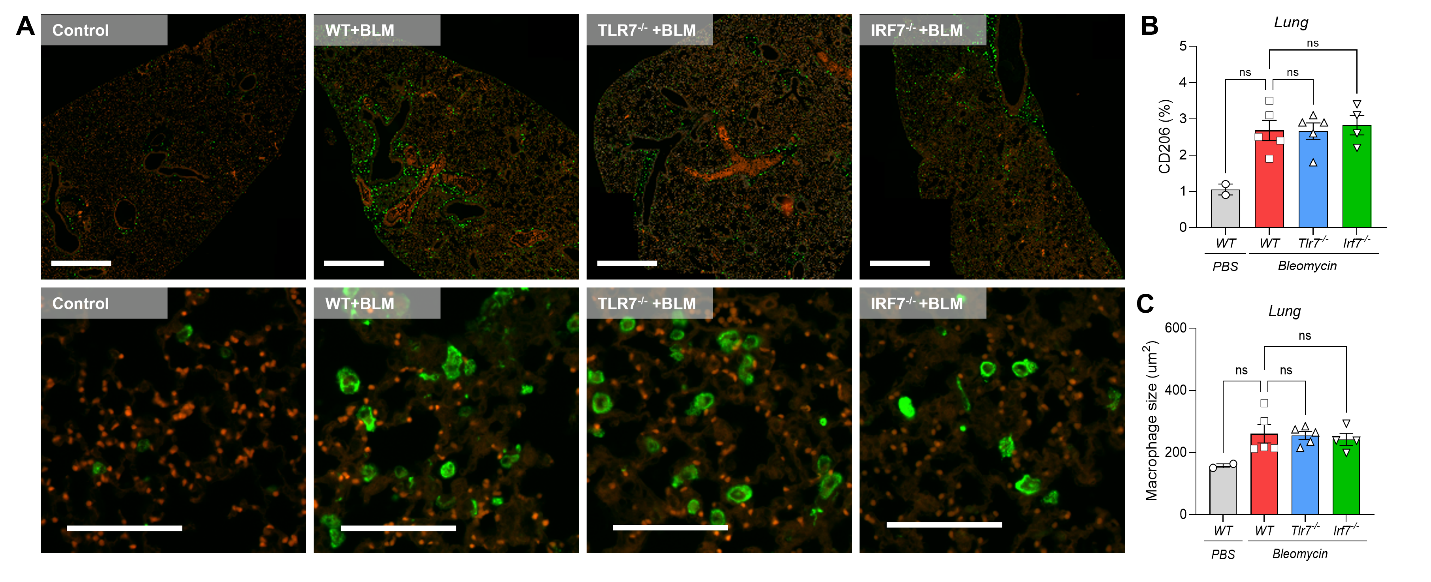
**

**Supplementary Figure 4. Lung immunofluorescence for CD206 and macrophage size quantification after bleomycin administration.** Mice were injected daily with bleomycin (BLM; 2.5 mg/kg) or PBS (Control) for 28 days to induce experimental systemic sclerosis. Lungs were collected on day 29 and processed for immunofluorescence. **(A)** Representative lung sections stained for CD206 (green) with tissue autofluorescence (orange). Upper panel: low magnification (scale bar = 500 μm). Lower panel: high magnification (scale bar = 100 μm). **(B)** Quantification of CD206+ area (%): no significant differences among groups. **(C)** Quantification of macrophage size (μm²): no significant differences were detected among groups. Data are shown as mean ± SEM with individual values. Group sizes: WT+PBS (n=2), WT+BLM (n=4), *Tlr7*^⁻/⁻^+BLM (n=4), and *Irf7*^⁻/⁻^+BLM (n=4). Statistical analysis was performed using one-way ANOVA followed by Tukey’s post hoc test. ns, not significant.

**
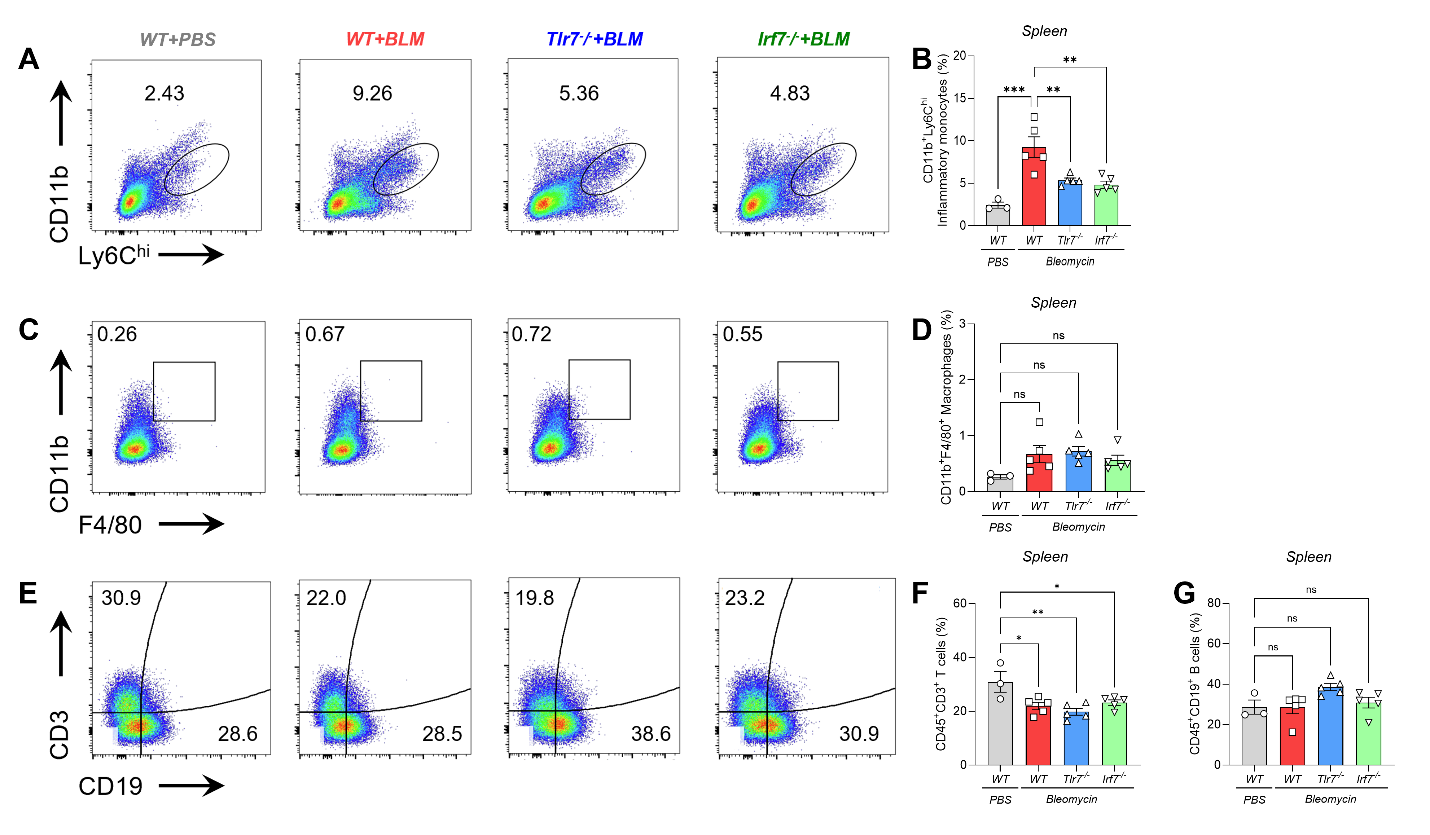
**

**Supplementary Figure 5. Immune cell composition in the spleen after bleomycin administration.** Mice were injected daily with bleomycin (BLM; 2.5 mg/kg) or PBS (Control) for 28 days to induce systemic sclerosis. Spleens were harvested on day 29 and processed for flow cytometry to quantify CD11b⁺Ly6C^hi^ inflammatory monocytes, CD11b⁺F4/80⁺ macrophages, CD45⁺CD3⁺ T cells, and CD45⁺CD19⁺ B cells. **(A)** Representative flow-cytometry dot plots of splenic CD11b⁺Ly6C^hi^ inflammatory monocytes. **(B)** Quantification of splenic CD11b⁺Ly6C^hi^ inflammatory monocytes: percentages were significantly reduced in PBS-treated WT and BLM-treated *Tlr7^–^/^–^* and *Irf7^–^/^–^* mice compared with BLM-treated WT mice. **(C)** Representative dot plots of splenic CD11b⁺F4/80⁺ macrophages. **(D)** Quantification of splenic CD11b⁺F4/80⁺ macrophages showed no significant differences across groups. **(E)** Representative dot plots of splenic CD45⁺CD3⁺ T cells and CD45⁺CD19⁺ B cells. **(F)** Quantification of CD45⁺CD3⁺ T cells revealed a significant reduction in BLM-treated WT, *Tlr7⁻/⁻*, and *Irf7⁻/⁻* mice compared with PBS-treated WT mice. **(G)** Quantification of CD45⁺CD19⁺ B cells showed no significant differences across groups. Data are shown as mean ± SEM and individual values. Group sizes: WT+PBS (n=3), WT+BLM (n=5), *Tlr7*^⁻/⁻^+BLM (n=5), and *Irf7*^⁻/⁻^+BLM (n=5). Statistical analysis was performed using one-way ANOVA followed by Tukey’s post hoc test. **p* < 0.05, ***p* < 0.01, ****p* < 0.001; ns, not significant.
